# An Enzymatic Flow-Based Preparative Route to Vidarabine

**DOI:** 10.3390/molecules25051223

**Published:** 2020-03-09

**Authors:** Lucia Tamborini, Clelia Previtali, Francesca Annunziata, Teodora Bavaro, Marco Terreni, Enrica Calleri, Francesca Rinaldi, Andrea Pinto, Giovanna Speranza, Daniela Ubiali, Paola Conti

**Affiliations:** 1 Department of Pharmaceutical Sciences, University of Milan, via Mangiagalli 25, 20133 Milano, Italy; clelia.previtali@unimi.it (C.P.); francesca.annunziata@unimi.it (F.A.); paola.conti@unimi.it (P.C.); 2 Department of Drug Sciences, University of Pavia, viale Taramelli 12, 27100 Pavia, Italy; teodora.bavaro@unipv.it (T.B.); marco.terreni@unipv.it (M.T.); enrica.calleri@unipv.it (E.C.); francesca.rinaldi01@universitadipavia.it (F.R.); 3 Department of Food, Environmental and Nutritional Sciences, University of Milan, via Celoria 2, 20133 Milano, Italy; andrea.pinto@unimi.it; 4 Department of Chemistry, University of Milan, via Golgi 19, 20133 Milano, Italy; giovanna.speranza@unimi.it

**Keywords:** flow bioreactor, enzyme immobilization, nucleoside phosphorylase, vidarabine, nucleosides

## Abstract

The bi-enzymatic synthesis of the antiviral drug vidarabine (arabinosyladenine, ara-A), catalyzed by uridine phosphorylase from *Clostridium perfringens* (*Cp*UP) and a purine nucleoside phosphorylase from *Aeromonas hydrophila* (*Ah*PNP), was re-designed under continuous-flow conditions. Glyoxyl–agarose and EziG^TM^1 (Opal) were used as immobilization carriers for carrying out this preparative biotransformation. Upon setting-up reaction parameters (substrate concentration and molar ratio, temperature, pressure, residence time), 1 g of vidarabine was obtained in 55% isolated yield and >99% purity by simply running the flow reactor for 1 week and then collecting (by filtration) the nucleoside precipitated out of the exiting flow. Taking into account the substrate specificity of *Cp*UP and *Ah*PNP, the results obtained pave the way to the use of the *Cp*UP/*Ah*PNP-based bioreactor for the preparation of other purine nucleosides.

## 1. Introduction

The use of biocatalysis in multiple industrial sectors is rapidly expanding due to the several benefits that it offers over traditional chemo-catalytic methods. Enzyme-catalyzed reactions are selective, safe, and environmentally friendly, thus meeting the increasing demand of industry for more efficient and sustainable processes [1,2]. However, low productivity and difficult product downstream are still the most often encountered bottlenecks in biocatalysis, which limit the implementation of enzymatic processes in industry [3]. Continuous flow technology is emerging as a key enabling tool for biocatalytic process intensification [4,5,6,7,8]. Flow processing has the potential to accelerate heterogeneous biotransformations due to biocatalyst high local concentration and enhanced mass transfer, making large-scale production more economically feasible in small equipment, with a substantial decrease in reaction time and improvement in space–time yield. Moreover, biocatalyst stability can benefit from an environment where harsh mixing is avoided. A further advantage of flow reactors is that their configuration can be easily customized to meet the specific requirements of the biotransformation. In this frame, the addition of in-line purification steps can assist the downstream process, also reducing time and costs associated with this often troublesome step. Methods based on the use of biotransformations in continuous flow reactors appear to be suitable for the intensification of industrially relevant processes, such as the synthesis of active pharmaceutical ingredients (APIs) [4,9].

Despite the enormous progress in nucleoside chemistry, the obtainment of nucleoside analogues is still a synthetic challenge [10,11]. Due to the exquisite selectivity of enzymes, the introduction of one or more biocatalytic steps in synthetic sequences has been demonstrated to simplify the preparation of these molecules, by cutting the number of reaction steps and avoiding by-product formation. Process intensification by flow reactor technology would be desirable to take another step forward in the full exploitation of enzyme-based reactions for the synthesis of nucleoside analogues.

Enzymes of nucleic acid metabolism, such as nucleoside phosphorylases (NPs, EC 2.4.2), have been conveniently used as biocatalysts in the synthesis of a number of nucleoside analogues [12,13,14,15,16]. These enzymes catalyze the reversible cleavage of the glycosidic bond of (deoxy)ribonucleosides in the presence of inorganic phosphate (Pi) to generate the nucleobase and α-D-(deoxy)ribose-1-phosphate (phosphorolysis). If a second nucleobase is added to the reaction medium, the formation of a new nucleoside can result (transglycosylation).

In this context, we focused our attention on the study of the flow biocatalyzed transglycosylation reaction to obtain nucleoside analogues of pharmaceutical interest, such as vidarabine (arabinosyladenine, ara-A). To achieve this, we used recombinant uridine phosphorylase from *Clostridium perfringens* (*Cp*UP) and a purine nucleoside phosphorylase from *Aeromonas hydrophila* (*Ah*PNP), an enzymatic twosome that has been successfully applied to the synthesis of both vidarabine (“one-pot, two-enzyme” transglycosylation) [17] and vidarabine-5′-monophosphate, in a batch mode (“one-pot, three-enzyme” transglycosylation and phosphorylation) [18].

## 2. Results and Discussion

**Scheme 1:**
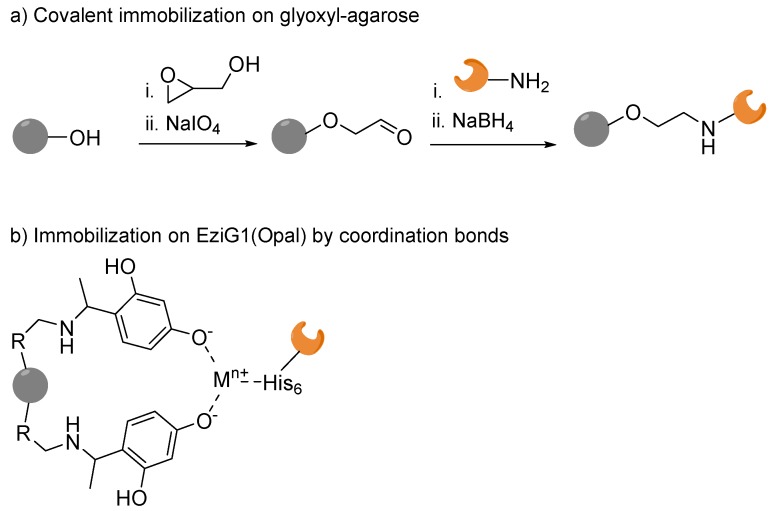
General scheme of the immobilization reactions discussed in the text. M^n+^ = metal ion of the CPG carrier (controlled pore glass) EziG^TM^1 (Opal) (e.g., Fe^3+^, Co^2+^); His_6_ = six-histidine tag.

With the aim to fully exploit the potential of the target biocatalyzed reaction in flow, we carried out a study on the co-immobilization of *Ah*PNP and *Cp*UP on two hydrophilic supports, i.e., glyoxyl–agarose (GA) and EziG^TM^1 (Opal) [19,20], which involve a different binding chemistry between the enzymes and the support (Scheme 1). Both carriers were packed into an Omnifit glass column and the enzymes were then immobilized by flowing a solution of the proteins through it. The obtained bioreactors, characterized in terms of immobilization yield and recovered activity, were used to perform the transglycosylation reaction in a continuous-flow mode. Parameters of the transglycosylation reaction (e.g., substrate concentration, molar ratio, temperature, pressure, residence time) have been screened for the highest yield and productivity. An array of substrates was tested to demonstrate the versatility of the synthetic approach and then to prepare the antiviral drug vidarabine on a gram scale. The manufacturing process was implemented by adding an in-line purification step for vidarabine to facilitate work-up and product recovery.

### 2.1. Immobilization on Glyoxyl–Agarose under Continuous Flow

The flow immobilization of each enzyme was first studied separately. *Cp*UP was immobilized first, reproducing the batch conditions previously reported [21]. To mimic the batch conditions (i.e., 3 h at room temperature), the buffer containing *Cp*UP (50 mM sodium carbonate pH 10, protein loading 5 mg per gram of carrier) was flowed for 3 h through the column (glass column i.d.: 6.6 mm; length: 100 mm) packed with glyoxyl–agarose (0.68 g, reactor volume: 0.68 mL) using a recirculation system; this step was followed by the chemical reduction of the newly formed imino bonds with a solution of NaBH_4_ recirculating for 30 min. Under these conditions, both yield and recovered activity of the immobilized protein were consistent with the results obtained in the in batch immobilization (100% and 35%, respectively). The activity of imm-*Cp*UP was determined spectrophotometrically by monitoring the phosphorolysis of 2′-deoxyuridine to uracil, after withdrawing the immobilized enzyme from the column. Then, the protocol was repeated by decreasing the immobilization time from 3 h to 1 h and the reduction step from 30 min to 15 min. The immobilized yield aligned with the previous results (100%), and the recovered activity was retained (34%).

Immobilization of *Ah*PNP resulted to be troublesome. In fact, under the same conditions as for *Cp*UP, the percentage of immobilized protein was less than a half (40%) and the recovered activity was only 14%. The immobilization was performed by recirculating a solution of the free enzyme (5 mg per gram of carrier) in 50 mM sodium carbonate buffer pH 10 containing hypoxanthine (5 mM) and glycerol (20% v/v). Again, the reduction of the imino bonds was performed with a solution of NaBH_4_ recirculating for 30 min.

Each immobilized enzyme recovered from the respective column was submitted to a stability test. Both *Cp*UP and *Ah*PNP were incubated for 24 h under mechanical stirring in 50 mM carbonate buffer at pH 10. The enzyme activity was then evaluated after 3, 6 and 24 h and, whereas imm-*Cp*UP resulted to be stable, imm-*Ah*PNP showed a loss of activity (about 85% after 24 h). Furthermore, both enzymes were incubated separately for 15 min at 95 °C in the presence of a denaturing agent, i.e., 2-mercaptoethanol; the stability of the immobilized enzymes was not affected in this case.

A flow protocol to evaluate the activity of the immobilized enzymes was also developed with the aim to have a straightforward method for assessing their activity without the need for “un-packing” the biocatalysts. In both cases, the phosphorolysis of the reference substrate (i.e., inosine for *Ah*PNP and 2′-deoxyuridine for *Cp*UP) was monitored to determine the residual activity upon immobilization. Therefore, a solution of inosine (20 mM) or a solution of 2′-deoxyuridine (20 mM) in 50 mM phosphate buffer pH 7.5 was pumped through the respective bioreactor and the exiting solution was collected and analyzed by HPLC. The percentage of phosphorolysis was calculated based on the depletion of the nucleoside, by monitoring the formation of the nucleobase (i.e., hypoxanthine and uracil, respectively; see Materials and Methods, Section 3.1).

Once the in-flow immobilization for each enzyme was evaluated, a protocol for the in-flow co-immobilization of the two enzymes was investigated. Given the issues concerning *Ah*PNP immobilization, we decided to immobilize it first, followed by *Cp*UP (protein loading: 7 mg of *Ah*PNP and 3 mg of *Cp*UP per gram of carrier), under the conditions reported above. From protein measurements of the exiting flow solution [22], immobilization yields resulted to be almost quantitative for *Cp*UP and 58% for *Ah*PNP. After chemical reduction, the recovered activity was 40% for *Cp*UP and 14% for *Ah*PNP.

### 2.2. Transglycosylation Reaction Using Glyoxyl–Agarose-Based CpUP/AhPNP-Bioreactor

**Scheme 2:**
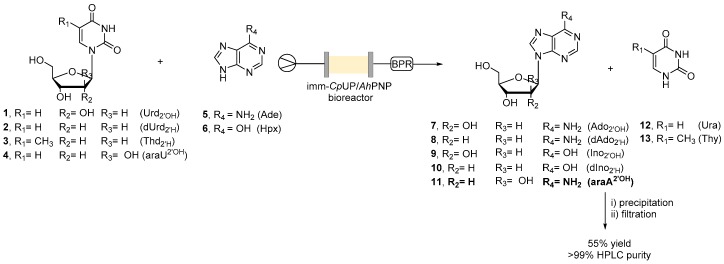
General scheme of the flow biocatalyzed transglycosylation reactions. BPR: backpressure regulator (20 psi). Legend: Urd_2′OH_ (**1**, uridine), dUrd_2′H_ (**2**, 2′-deoxyuridine), Thd_2′H_ (**3**, thymidine), araU^2′OH^ (**4**, arabinofuranosyluracil), Ade (**5**, adenine), Hpx (**6**, hypoxanthine), Ado_2′OH_ (**7**, adenosine), dAdo_2′H_ (**8**, 2′-deoxyadenosine), Ino_2′OH_ (**9**, inosine), dIno_2′H_ (**10**, 2′-deoxyinosine), araA^2′OH^ (**11**, arabinosyladenine, vidarabine), Ura (**12**, uracil), Thy (**13**, thymine).

The *Cp*UP/*Ah*PNP -bioreactor (reactor volume: 0.68 mL) was tested in the synthesis of adenosine (**7**, Scheme 2) by transglycosylation reaction using uridine (**1**) as the sugar donor (4 mM) and adenine (**5**) as the nucleobase (2 mM) in 50 mM phosphate buffer pH = 7.5. These experimental conditions (i.e., buffer, substrate concentration and ratio, pH) were selected from previously reported studies in batch [21]. The solution containing the sugar donor and the sugar acceptor were pumped through the bioreactor pressurized at 20 psi.

Different residence times and substrate concentrations were screened (Table 1). In only 5 min of residence time, an 85% conversion was achieved, as determined by HPLC (Table 1, entry 1). Then, to increase the productivity of the process, the concentration of the sugar donor was set to 100 mM, keeping constant the 2:1 ratio to the sugar acceptor. Interestingly, also at the highest tested concentration (Table 1, entry 10), an 89% conversion was obtained in only 5 min. However, due to the poor water solubility of the substrates, the addition of a co-solvent (i.e., DMF) in the range 10–20% *v/v* was necessary (see Table 1). DMF was selected as the co-solvent according to the stability studies previously carried out in the batch mode transglycosylation [17]. An increase of the residence time up to 20 min (Table 1, entries 3 and 4) did not lead to a higher conversion. Then, using the highest concentration that does not require any co-solvent (i.e., 16 mM uridine, 8 mM adenine), a further decrease of the residence time was evaluated. The reaction reached the equilibrium after only 1 min of residence time (Table 1, entry 7). Finally, an increase of the temperature (up to 38 °C) and different molar ratios between the sugar donor and the sugar acceptor (1:1 and 4:1) were also tested but, in all cases, no beneficial effects were observed (data not shown).

The stability of the bioreactor under continuous work was tested in the conditions reported in Table 1, entry 7 (reactor volume: 0.68 mL; residence time: 1 min; T = 28 °C; P = 20 psi; 16 mM uridine (**1**); 8 mM adenine (**5**); flow stream: 50 mM phosphate buffer pH 7.5) and the exiting flow stream was collected over 5 days (collected volume ~5 L), monitoring the reaction outcome by HPLC every 6 h. HPLC analysis showed that the enzymes retained their activity (the conversion was constant, approximately 85%) even after 5 days of continuous work. After that time, the flow stream was stopped. This result demonstrates the excellent stability of the new bioreactor under the applied operational conditions (Table 2). Continuous flow synthesis of adenosine (**7**) resulted in a good space time yield (1.8 g/day) and a biocatalyst productivity of 0.98 mmol_product_/mg_enzyme_ (defined as mmol of product formed over 24 h per amount of biocatalyst).

The versatility of the bioreactor was evaluated using different sugar donors and sugar acceptors under the conditions set-up for uridine/adenine transglycosylation reaction (Table 3), resulting in conversions between 40 and 76%.

### 2.3. Co-Immobilization on EziG^TM^ Carriers in Continuous Flow

EziG^TM^ is a support made of controlled porosity glass particles, which offers excellent flow through properties due to its interconnecting pore structure and incompressible/non-swelling nature [19,20]. The particles contain chelated Fe(III) for His-tag binding. Enzymes bind the carrier through a coordination bond. EziG^TM^1 (Opal), a hydrophilic carrier, was selected for this study. The flow co-immobilization (glass column i.d.: 10 mm, length: 100 mm; EziG^TM^1: 950 mg; reactor volume: 3.0 mL) was almost quantitative (97%) in about 30 min of residence time, resulting in good recovered activities (22% for *Cp*UP and 51% for *Ah*PNP). Excellent results were obtained in the model transglycosylation reaction with uridine/adenine, achieving an 84% conversion in only 1 min of residence time.

### 2.4. Continuous Flow Synthesis of Ara-A (11) and Product Isolation

**Figure 1:**
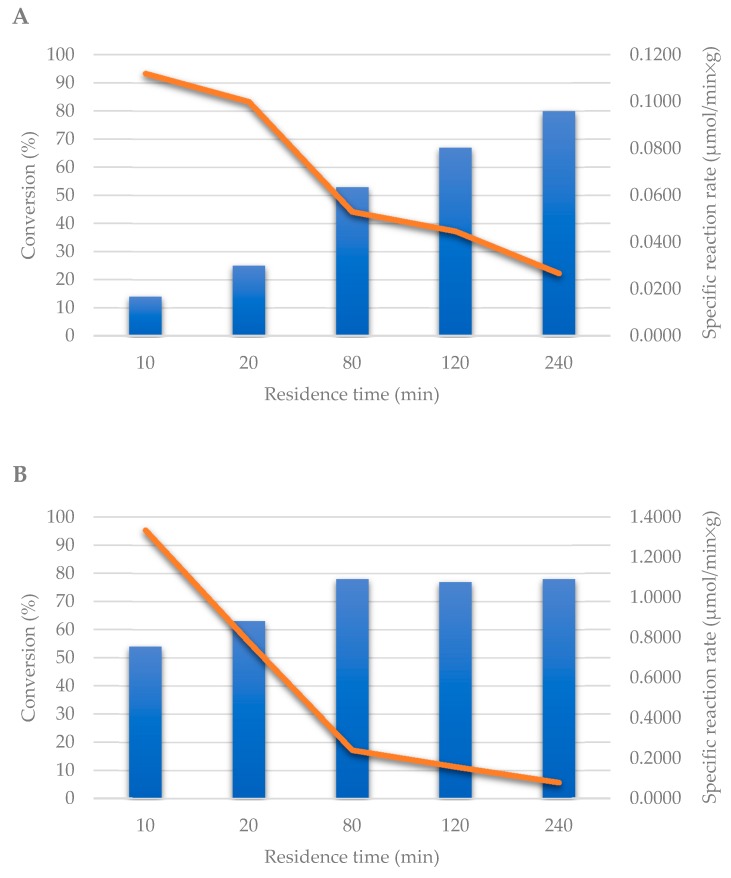
Screening of the residence time for the synthesis of ara-A (vidarabine, **11**). (**A**) enzymes immobilized on glyoxyl–agarose; (**B**) enzymes immobilized on EziG^TM^1. Experimental conditions: [araU] = 16 mM; [Ade] = 8 mM; 50 mM phosphate buffer pH 7.5; T = 28 °C; P = 20 psi. Conversions (%; blue bars) were determined by HPLC (see Materials and Methods for details). Specific reaction rates (µmol min^−1^ g^−1^; orange line) were calculated using the equation: r = [P] × f/m_e_, where [P] is the concentration of the product (μmol mL^−1^), f is the flow rate (mL min^−1^) and m_e_ is the amount of immobilized enzyme (g) [23].

In order to synthesize ara-A (**11**), both the glyoxyl–agarose and the EziG^TM^1-based bioreactors were tested. A stock solution containing the sugar donor (araU, **4**, 16 mM) and the sugar acceptor (adenine, **5**, 8 mM) was prepared avoiding any use of organic co-solvent, thus increasing the “greenness” of the protocol and simplifying the product recovery. Residence time was modified with the aim to achieve the highest conversion (Figure 1). Using the glyoxyl–agarose-based bioreactor, 80% conversion was reached after 4 h of residence time, whereas, using the EziG^TM^1-based bioreactor, a similar conversion was obtained in only 80 min.

Then, the stability of the EziG^TM^1 bioreactor was tested under continuous work, monitoring the reaction outcome every 2 h by HPLC. The conversion rapidly (after 24 h) dropped to less than 10% and protein was detected in the exiting flow stream. Therefore, in the present study, glyoxyl–agarose was selected as carrier and the bioreactor was used for the continuous flow synthesis of ara-A (**11**). To this aim, a larger reactor was prepared (glass column i.d.: 15 mm, length: 150 mm; glyoxyl–agarose: 10 g; reactor volume: 10 mL) and 1 L of the solution containing the sugar donor and the sugar acceptor was submitted to the biotransformation, setting the residence time at 120 min (67% conversion). The poor water solubility of araA was exploited for product recovery. In detail, the exiting flow stream was directly collected into a cooled vessel to favor the product precipitation. Vidarabine was then recovered by filtration under vacuum, washed with cooled water and dried under vacuum. The reactor was left working for 8 days and then the product was recovered in 55% isolated yield and very high chemical purity (>99%), as determined by HPLC and NMR. Interestingly, even after 8 days of continuous work, the conversion was still about 65%.

## 3. Materials and Methods

All reagents were purchased from Merck Sigma-Aldrich (Milan, Italy). Agarose gel 6B-CL was activated to glyoxyl–agarose as previously reported [24]. TLC analyses were performed using commercial silica gel 60 F254 aluminum sheets. HPLC analyses were performed using a Waters 1525 Binary HPLC Pump, equipped with a Waters 2489 UV–vis detector (Waters, Milford, MA, USA) and an Ascenti C18 column (25 cm × 4 mm, 4 μm particle size). The Bradford assay and *Cp*UP activity assay were performed using the spectrophotometer Ultrospec 1000 (Pharmacia Biotech, Cologno Monzese, Italy). ^1^H-NMR of ara-A (**11**) in DMSO*d6* was recorded on a Varian Gemini 300 MHz spectrometer (Varian, Palo Alto, CA, USA). The continuous flow reactions were performed using either a R2+/R4 series or an E series flow reactor, commercially available from Vapourtec (Cambridge, UK) equipped with Omnifit glass columns with one fixed and one adjustable endfits. Recombinant *Cp*UP and *Ah*PNP were prepared as previously reported [17]. One international unit (IU) corresponds to the amount of enzyme that transforms 1 μmol of substrate per minute under specific temperature and pH values, while the specific activity is defined as units of enzyme activity per milligram of protein.

### 3.1. Analytical Methods

Samples for the standard activity assay of *Ah*PNP (Section 3.6), flow activity assay of *Ah*PNP (Section 3.7), flow activity assay of *CpUP* (Section 3.8) and flow transglycosylation reaction (Section 3.9) were prepared as reported below. Samples were analyzed by HPLC as follows: injection volume: 10 µL; mobile phase: H_2_O/MeOH 9:1; flow rate: 1.0 mL min^−1^; λ: 260 nm. Retention times (t_R_): thymine (**13**) = 6.6 min; thymidine (**3**) = 13.2 min; adenine (**5**) = 8.9 min; adenosine (**7**) = 27.8 min; 2′-deoxyadenosine (**8**) = 26.4 min; uracil (**12**) = 3.6 min; uridine (**1**) = 4.8 min; 2′-deoxyuridine (**2**) = 7.1 min; 2′-deoxyinosine (**10**) = 10.9 min; inosine (**9**) = 4.7 min; hypoxanthine (**6**) = 8.2 min. The percentage of conversion was calculated on the basis of the depletion of the sugar acceptor (nucleobase) and monitoring the formation of the nucleoside product: Conversion [%] = [product area/(product area + base area)] × 100.

### 3.2. Immobilization Yields

The immobilization yields (%) were determined as: immobilized protein and activity recovery as reported by Sheldon et al. [25] according to the following equations: immobilized protein (%): (immobilized protein/loaded protein) × 100; activity recovery (%): (observed activity of the immobilized enzyme/starting activity) × 100.

### 3.3. Co-Immobilization of CpUP and AhPNP on Glyoxyl–Agarose under Flow Conditions

Glyoxyl–agarose (0.68 g, volume 0.68 mL) was packed into an Omnifit glass column (6.6 mm × 100 mm) and the packed-bed reactor was washed with 50 mM carbonate buffer pH 10. A mixture of glycerol (20% v/v) and hypoxanthine (5 mM) in 50 mM carbonate buffer pH 10 (4.4 mL) was prepared. In order to dissolve hypoxanthine, the suspension was sonicated using a VWR ultrasonic bath (3 × 10 min, frequency: 35 Khz). To the obtained solution, 4.8 mg of *Ah*PNP (156 μL) were added. The solution was flowed through the column at a flow rate of 0.1 mL min^−1^; the exiting solution was collected and recirculated through the column for 3 h. The residual protein was determined by Bradford assay [22]. Then, a solution of 2.0 mg of *Cp*UP (250 μL) in 50 mM carbonate buffer pH 10 (3 mL) was prepared and flowed through the same column at a flow rate of 0.1 mL min^−1^. The exiting solution was collected and recirculated for 3 h. The residual protein was determined by Bradford assay [22]. Chemical reduction of imines was carried out by adding NaBH_4_ (14 mg) to the recirculating mixture at 0.2 mL min^−1^ for 30 min. The bioreactor was then washed with water for 30 min at 0.4 mL min^−1^.

### 3.4. Co-Immobilization of CpUP and AhPNP on EziG^TM^1 (Opal) under Flow Conditions

EziG^TM^1 (950 mg, 3.0 mL) was packed into an Omnifit glass column (10 mm × 100 mm). A solution of 25 mg of *Cp*UP stock solution (6.16 mg mL^−1^) and 25 mg of *Ah*PNP stock solution (22.4 mg mL^−1^) in 20 mM phosphate buffer pH 7.5 and 300 mM NaCl solution was prepared (16.7 mL). The solution was flowed at 0.1 mL min^−1^ and recirculated for 2 h. The residual protein was determined by the Bradford assay [22].

### 3.5. Standard Activity Assay of CpUP

The activity of soluble *Cp*UP toward 2′-deoxyuridine (**2**) was determined as previously reported [21]. Briefly, a solution of 2′-deoxyuridine (**2**) in 50 mM potassium phosphate buffer pH 7.5 (230 μL) and deionized water (645 μL) was prepared. After the addition of the soluble (5 μL of enzyme preparation diluted 1:10) or immobilized (10 mg) enzyme, the mixture was incubated under mechanical stirring. At different times (5 and 10 min), samples were withdrawn and 10 M NaOH (70 μL) was added. The increase in absorbance at 297 nm was then measured.

### 3.6. Standard Activity Assay of AhPNP

The activity of soluble *Ah*PNP was assessed by measuring the phosphorolysis of inosine (**9**) as previously reported [21]. Briefly, to a 5 mM solution of inosine (**9**) in 50 mM phosphate buffer pH 7.5, the soluble enzyme (30 μL of enzyme diluted 1:10) was added and the mixture was incubated under mechanical stirring. At different times (5 and 10 min), 500 μL of the solution were taken and mixed with 500 μL of MeOH. The mixture was heated for 5 min at 95 °C and then centrifuged (10 min, 13000 rpm). The supernatant was taken and diluted with a solution of H_2_O/MeOH (9:1), in order to obtain 1 mM solution. The sample was then analyzed by HPLC. For the immobilized *Ah*PNP, the same assay was performed by using 20 mg of the immobilized biocatalyst under magnetic stirring. At different times (5 and 10 min), 500 μL of the solution were taken and filtered. The sample was then diluted with a solution of H_2_O/MeOH (9:1) in order to obtain a 1 mM solution. The sample was then analyzed by HPLC.

### 3.7. Flow Activity Assay of AhPNP

A solution of inosine (**9**, 5 mL, 20 mM) in 50 mM phosphate buffer pH 7.5 was prepared and pumped through the column packed with immobilized *Ah*PNP. The outcome of the reaction at different residence times (i.e., 1, 2.5, 5 and 10 min) was analyzed by HPLC.

### 3.8. Flow Activity Assay of CpUP

A solution of 2′-deoxyuridine (**2**, 5 mL, 20 mM) in 50 mM phosphate buffer pH 7.5 was prepared and pumped through the column packed with immobilized *Cp*UP. The outcome of the reaction at different residence times (i.e., 1, 2.5, 5 and 10 min) was analyzed by HPLC.

### 3.9. General Procedure for the Flow Transglycosylation Reaction

A solution of the sugar donor and the sugar acceptor was prepared in 50 mM phosphate buffer pH 7.5. In the case of hypoxanthine (**6**), in order to dissolve it, the suspension was sonicated (3 × 10 min). The temperature was set at 28 °C. The column packed with immobilized *Cp*UP and *Ah*PNP (0.68 g, 0.68 mL) was washed with 50 mM phosphate buffer pH 7.5 at 0.2 mL min^−1^ for 10 min at atmospheric pressure. The substrate solution (2.0 mL) was flowed through the bioreactor. Residence times and concentrations were varied as reported in Table 1. The reaction outcome was monitored by HPLC. A sample of the exiting flow stream (200 µL) was diluted with a mixture of H_2_O/MeOH (9:1), in order to obtain a sample concentration of 0.1 mM, and was used for the analysis.

### 3.10. Synthesis of Vidarabine (**11**)

A solution of arabinofuranosyluracil (**4**, 16 mM) and adenine (**5**, 8 mM) was prepared in 50 mM phosphate buffer pH 7.5 (1 L). The column (15 mm × 150 mm) packed with immobilized *Cp*UP and *Ah*PNP (10 g, 10 mL) was washed with 50 mM phosphate buffer pH 7.5 at 1.0 mL min^−1^ for 30 min. The substrate solution was flowed into the system at 83 µL min^−1^ (residence time: 120 min), at 28 °C and 20 psi for 8 days. The reaction outcome was monitored by HPLC by collecting a sample of the exiting flow stream every 8 h. The exiting solution was collected into a flask, which was then cooled at 4 °C to favor the product precipitation. The solid was filtered under vacuum, washed with cooled water (50 mL) and dried under vacuum. Vidarabine (**11**) was isolated in 55% yield as a white solid (1.1 g). ^1^H-NMR was in agreement with literature data [17].

## 4. Conclusions

The combination of biocatalysis and flow chemistry technology is recognized as an efficient tool for process intensification, as demonstrated by more and more successful examples of industrially relevant processes.

In this frame, we have set-up a biocatalyzed synthesis for the preparation of the antiviral drug vidarabine (**11**), using a bioreactor in flow. The bioreactor was prepared by in-flow co-immobilization of *Cp*UP and *Ah*PNP on glyoxyl–agarose. The synthesis was scaled-up to gram scale: 1 g of vidarabine was synthesized and purified in an overall time of 8 days. The system is versatile and, in principle, transferable to the preparation of other nucleoside analogues. Further scale-up could be achieved by simply letting the reactor flow for an extended period of time, without modifying, in any way, the reaction set-up, or by increasing the size of the bioreactor, or again, by working with parallel reactors.

## Figures and Tables

**Table 1:** Synthesis of adenosine (**7**) by continuous flow transglycosylation reaction catalyzed by co-immobilized *Cp*UP and *Ah*PNP: screening of the reaction conditions.

Entry	Residence Time (min)	[Urd, 1] (mM)	[Ade, 5] (mM)	Conversion (%) ^a^
1	5	4	2	85
2	5	8	4	86
3	10	8	4	84
4	20	8	4	84
5	5	16	8	86
6	2.5	16	8	86
7	1	16	8	86
8 ^b^	5	32	16	88
9 ^b^	5	40	20	89
10 ^c^	5	100	50	89

^a^ Determined by HPLC: [product area/(product area + substrate area)] × 100. Conversions (%) are the average of two experiments. ^b^ DMF (10% *v/v*) was added as the co-solvent. ^c^ DMF (20% *v/v*) was added as the co-solvent. Experimental conditions: 50 mM phosphate buffer pH 7.5; T = 28 °C; P = 20 psi.

**Table 2:** Synthesis of adenosine (**7**) by continuous flow transglycosylation reaction catalyzed by co-immobilized *Cp*UP and *Ah*PNP: system productivity.

Residence Time (min)	Conversion (%)	Space Time Yield (g/day)	Catalyst Productivity (24 h) (mmol_product_/mg_enzyme_)
1	85	1.8	0.98

Conversion (%) was monitored over 5 days by sampling the exiting flow stream every 6 h. Each analysis was repeated twice.

**Table 3:** Synthesis of purine ribo- and 2′-deoxyribonucleosides (**7**–**10**) by a continuous flow transglycosylation reaction catalyzed by co-immobilized *Cp*UP and *Ah*PNP.

Sugar Donor	Sugar Acceptor	Product	Conversion ^a^ (%)
**1** (Urd)	**5** (Ade)	**7** (Ado)	76
**1** (Urd)	**6** (Hpx)	**9** (Ino)	45
**2** (dUrd)	**5** (Ade)	**8** (dAdo)	70
**2** (dUrd)	**6** (Hpx)	**10** (dIno)	50
**3** (Thd)	**5** (Ade)	**8** (dAdo)	73
**3** (Thd)	**6** (Hpx)	**10** (dIno)	40

^a^ Determined by HPLC: [product area/(product area + substrate area)] × 100. Conversions (%) are the average of two experiments. Experimental conditions: 50 mM phosphate buffer pH 7.5; [sugar donor] = 16 mM; [sugar acceptor] = 8 mM; residence time = 1 min; T = 28 °C; P = 20 psi.
